# Correction: Cognition, physical function, and life purpose in the rural elderly population: A systematic review protocol

**DOI:** 10.1371/journal.pone.0310260

**Published:** 2024-09-06

**Authors:** Hércules Lázaro Morais Campos, Elisa Brosina De Leon, Ingred Merllin Batista de Souza, Anna Quialheiro, Elizabete Regina Araújo de Oliveira

The following information is missing from the funding statement: The publication costs were covered by funding from the Espírito Santo Research and Innovation Support Foundation (FAPES).

## References

[1] Morais Campos HL, De Leon EB, Merllin Batista de Souza I, Quialheiro A, Araújo de Oliveira ER(2024) Cognition, physical function, and life purpose in the rural elderly population: A systematic review protocol. PLoS ONE 19(6): e0291699. 10.1371/journal.pone.0291699 38861545 PMC11166331

