# Supplementary material for: Cognition, physical function, and life purpose in the rural elderly population: A systematic review protocol
Source: PLoS One. 2024 Jun 11;19(6):e0291699. doi: 10.1371/journal.pone.0291699 (PMC11166331; doi:10.1371/journal.pone.0291699)
Supplement: S1 Appendix — (DOCX) [file pone.0291699.s002.docx]

**Appendix 1: Complete Research Strategy**

**PubMED**

"Physical functioning" OR "Cognition" OR "Cognitions" OR "Cognitive Function" OR "Cognitive Functions" OR "Function, Cognitive" OR "Functions, Cognitive" OR "Life purpose") AND ("Aged" OR "Elderly" OR "Old" OR "Rural aging") AND ("Rural Population" OR "Communities, Rural" OR "Community, Rural" OR "Distribution, Rural Spatial" OR "Distributions, Rural Spatial" OR "Medium Communities" OR "Population, Rural" OR "Populations, Rural" OR "Rural Communities" OR "Rural Community" OR "Rural Populations" OR "Rural Settlement" OR "Rural Settlements" OR "Rural Spatial Distribution" OR "Rural Spatial Distributions" OR "Small Communities" OR "Small Community"

"Physical functioning"[Title/Abstract] OR "Cognition"[Title/Abstract] OR "Cognitions"[Title/Abstract] OR "Cognitive Function"[Title/Abstract] OR "Cognitive Functions"[Title/Abstract] OR "Function, Cognitive"[Title/Abstract] OR "Functions, Cognitive"[Title/Abstract] OR "Life purpose"[Title/Abstract]) AND ("Aged"[Title/Abstract] OR "Elderly"[Title/Abstract] OR "Old"[Title/Abstract] OR "Rural aging"[Title/Abstract]) AND ("Rural Population"[Title/Abstract] OR "Communities, Rural"[Title/Abstract] OR "Community, Rural"[Title/Abstract] OR "Distribution, Rural Spatial"[Title/Abstract] OR "Distributions, Rural Spatial"[Title/Abstract] OR "Medium Communities"[Title/Abstract] OR "Population, Rural"[Title/Abstract] OR "Populations, Rural"[Title/Abstract] OR "Rural Communities"[Title/Abstract] OR "Rural Community"[Title/Abstract] OR "Rural Populations"[Title/Abstract] OR "Rural Settlement"[Title/Abstract] OR "Rural Settlements"[Title/Abstract] OR "Rural Spatial Distribution"[Title/Abstract] OR "Rural Spatial Distributions"[Title/Abstract] OR "Small Communities"[Title/Abstract] OR "Small Community"[Title/Abstract]

"Personal satisfaction" OR "Life purpose" OR "Goals" OR "Satisfaction" OR "Satisfaction, Personal" OR "Subjective Well-Being") AND ("Cognition" OR "Cognitions" OR "Cognitive Function" OR "Cognitive Functions" OR "Function, Cognitive" OR "Functions, Cognitive") AND ("Disability" OR "Physical functioning" OR "functional ability") AND ("Aged" OR "Elderly" OR "Old" OR "Rural aging") AND ("Rural Population" OR "Communities, Rural" OR "Community, Rural" OR "Distribution, Rural Spatial" OR "Distributions, Rural Spatial" OR "Medium Communities" OR "Population, Rural" OR "Populations, Rural" OR "Rural Communities" OR "Rural Community" OR "Rural Populations" OR "Rural Settlement" OR "Rural Settlements" OR "Rural Spatial Distribution" OR "Rural Spatial Distributions" OR "Small Communities" OR "Small Community"

**Scopus**

ALL("Physical functioning" OR "Cognition" OR "Cognitions" OR "Cognitive Function" OR "Cognitive Functions" OR "Function, Cognitive" OR "Functions, Cognitive" OR "Life purpose") AND ALL("Aged" OR "Elderly" OR "Old" OR "Rural aging") AND ALL("Rural Population" OR "Communities, Rural" OR "Community, Rural" OR "Distribution, Rural Spatial" OR "Distributions, Rural Spatial" OR "Medium Communities" OR "Population, Rural" OR "Populations, Rural" OR "Rural Communities" OR "Rural Community" OR "Rural Populations" OR "Rural Settlement" OR "Rural Settlements" OR "Rural Spatial Distribution" OR "Rural Spatial Distributions" OR "Small Communities" OR "Small Community")

ALL("Physical functioning" OR "Cognition" OR "Cognitions" OR "Cognitive Function" OR "Cognitive Functions" OR "Function, Cognitive" OR "Functions, Cognitive" OR "Life purpose") AND ALL("Aged" OR "Elderly" OR "Old" OR "Rural aging") AND ALL("Rural Population" OR "Communities, Rural" OR "Community, Rural" OR "Distribution, Rural Spatial" OR "Distributions, Rural Spatial" OR "Medium Communities" OR "Population, Rural" OR "Populations, Rural" OR "Rural Communities" OR "Rural Community" OR "Rural Populations" OR "Rural Settlement" OR "Rural Settlements" OR "Rural Spatial Distribution" OR "Rural Spatial Distributions" OR "Small Communities" OR "Small Community") AND ALL("Meta-Analysis" OR "Multicenter Study" OR "Observational Study" OR "Review" OR "Systematic Review")

ALL("Physical functioning" OR "Cognition" OR "Cognitions" OR "Cognitive Function" OR "Cognitive Functions" OR "Function, Cognitive" OR "Functions, Cognitive" OR "Life purpose") AND ALL("Aged" OR "Elderly" OR "Old" OR "Rural aging") AND ALL("Rural Population" OR "Communities, Rural" OR "Community, Rural" OR "Distribution, Rural Spatial" OR "Distributions, Rural Spatial" OR "Medium Communities" OR "Population, Rural" OR "Populations, Rural" OR "Rural Communities" OR "Rural Community" OR "Rural Populations" OR "Rural Settlement" OR "Rural Settlements" OR "Rural Spatial Distribution" OR "Rural Spatial Distributions" OR "Small Communities" OR "Small Community") AND ALL("Meta-Analysis" OR "Multicenter Study" OR"Observational Study" OR "Review" OR "Systematic Review" OR "Cross-Sectional Studies")

TITLE-ABS("Physical functioning" OR "Cognition" OR "Cognitions" OR "Cognitive Function" OR "Cognitive Functions" OR "Function, Cognitive" OR "Functions, Cognitive" OR "Life purpose") AND TITLE-ABS("Aged" OR "Elderly" OR "Old" OR "Rural aging") AND TITLE-ABS("Rural Population" OR "Communities, Rural" OR "Community, Rural" OR "Distribution, Rural Spatial" OR "Distributions, Rural Spatial" OR "Medium Communities" OR "Population, Rural" OR "Populations, Rural" OR "Rural Communities" OR "Rural Community" OR "Rural Populations" OR "Rural Settlement" OR "Rural Settlements" OR "Rural Spatial Distribution" OR "Rural Spatial Distributions" OR "Small Communities" OR "Small Community") AND ALL("Meta-Analysis" OR "Multicenter Study" OR"Observational Study" OR "Review" OR "Systematic Review" OR "Cross-Sectional Studies")

ALL("Personal satisfaction" OR "Life purpose" OR "Goals" OR "Satisfaction" OR "Satisfaction, Personal" OR "Subjective Well-Being") AND ALL("Cognition" OR "Cognitions" OR "Cognitive Function" OR "Cognitive Functions" OR "Function, Cognitive" OR "Functions, Cognitive") AND ALL("Disability" OR "Physical functioning" OR "functional ability") AND ALL("Aged" OR "Elderly" OR "Old" OR "Rural aging") AND ALL("Rural Population" OR "Communities, Rural" OR "Community, Rural" OR "Distribution, Rural Spatial" OR "Distributions, Rural Spatial" OR "Medium Communities" OR "Population, Rural" OR "Populations, Rural" OR "Rural Communities" OR "Rural Community" OR "Rural Populations" OR "Rural Settlement" OR "Rural Settlements" OR "Rural Spatial Distribution" OR "Rural Spatial Distributions" OR "Small Communities" OR "Small Community")

**Web Of Science**

ALL=("Physical functioning" OR "Cognition" OR "Cognitions" OR "Cognitive Function" OR "Cognitive Functions" OR "Function, Cognitive" OR "Functions, Cognitive" OR "Life purpose") AND ALL=("Aged" OR "Elderly" OR "Old" OR "Rural aging") AND ALL=("Rural Population" OR "Communities, Rural" OR "Community, Rural" OR "Distribution, Rural Spatial" OR "Distributions, Rural Spatial" OR "Medium Communities" OR "Population, Rural" OR "Populations, Rural" OR "Rural Communities" OR "Rural Community" OR "Rural Populations" OR "Rural Settlement" OR "Rural Settlements" OR "Rural Spatial Distribution" OR "Rural Spatial Distributions" OR "Small Communities" OR "Small Community")

ALL=("Physical functioning" OR "Cognition" OR "Cognitions" OR "Cognitive Function" OR "Cognitive Functions" OR "Function, Cognitive" OR "Functions, Cognitive" OR "Life purpose") AND ALL=("Aged" OR "Elderly" OR "Old" OR "Rural aging") AND ALL=("Rural Population" OR "Communities, Rural" OR "Community, Rural" OR "Distribution, Rural Spatial" OR "Distributions, Rural Spatial" OR "Medium Communities" OR "Population, Rural" OR "Populations, Rural" OR "Rural Communities" OR "Rural Community" OR "Rural Populations" OR "Rural Settlement" OR "Rural Settlements" OR "Rural Spatial Distribution" OR "Rural Spatial Distributions" OR "Small Communities" OR "Small Community") AND ALL=("Meta-Analysis" OR "Multicenter Study" OR "Observational Study" OR "Review" OR "Systematic Review")

ALL=("Physical functioning" OR "Cognition" OR "Cognitions" OR "Cognitive Function" OR "Cognitive Functions" OR "Function, Cognitive" OR "Functions, Cognitive" OR "Life purpose") AND ALL=("Aged" OR "Elderly" OR "Old" OR "Rural aging") AND ALL=("Rural Population" OR "Communities, Rural" OR "Community, Rural" OR "Distribution, Rural Spatial" OR "Distributions, Rural Spatial" OR "Medium Communities" OR "Population, Rural" OR "Populations, Rural" OR "Rural Communities" OR "Rural Community" OR "Rural Populations" OR "Rural Settlement" OR "Rural Settlements" OR "Rural Spatial Distribution" OR "Rural Spatial Distributions" OR "Small Communities" OR "Small Community") AND ALL=("Meta-Analysis" OR "Multicenter Study" OR"Observational Study" OR "Review" OR "Systematic Review" OR "Cross-Sectional Studies")

(TI=("Physical functioning" OR "Cognition" OR "Cognitions" OR "Cognitive Function" OR "Cognitive Functions" OR "Function, Cognitive" OR "Functions, Cognitive" OR "Life purpose") AND TI=("Aged" OR "Elderly" OR "Old" OR "Rural aging") AND TI=("Rural Population" OR "Communities, Rural" OR "Community, Rural" OR "Distribution, Rural Spatial" OR "Distributions, Rural Spatial" OR "Medium Communities" OR "Population, Rural" OR "Populations, Rural" OR "Rural Communities" OR "Rural Community" OR "Rural Populations" OR "Rural Settlement" OR "Rural Settlements" OR "Rural Spatial Distribution" OR "Rural Spatial Distributions" OR "Small Communities" OR "Small Community")) OR (AB=("Physical functioning" OR "Cognition" OR "Cognitions" OR "Cognitive Function" OR "Cognitive Functions" OR "Function, Cognitive" OR "Functions, Cognitive" OR "Life purpose") AND AB=("Aged" OR "Elderly" OR "Old" OR "Rural aging") AND AB=("Rural Population" OR "Communities, Rural" OR "Community, Rural" OR "Distribution, Rural Spatial" OR "Distributions, Rural Spatial" OR "Medium Communities" OR "Population, Rural" OR "Populations, Rural" OR "Rural Communities" OR "Rural Community" OR "Rural Populations" OR "Rural Settlement" OR "Rural Settlements" OR "Rural Spatial Distribution" OR "Rural Spatial Distributions" OR "Small Communities" OR "Small Community"))

(TI=("Physical functioning" OR "Cognition" OR "Cognitions" OR "Cognitive Function" OR "Cognitive Functions" OR "Function, Cognitive" OR "Functions, Cognitive" OR "Life purpose") AND TI=("Aged" OR "Elderly" OR "Old" OR "Rural aging") AND TI=("Rural Population" OR "Communities, Rural" OR "Community, Rural" OR "Distribution, Rural Spatial" OR "Distributions, Rural Spatial" OR "Medium Communities" OR "Population, Rural" OR "Populations, Rural" OR "Rural Communities" OR "Rural Community" OR "Rural Populations" OR "Rural Settlement" OR "Rural Settlements" OR "Rural Spatial Distribution" OR "Rural Spatial Distributions" OR "Small Communities" OR "Small Community")) OR (AB=("Physical functioning" OR "Cognition" OR "Cognitions" OR "Cognitive Function" OR "Cognitive Functions" OR "Function, Cognitive" OR "Functions, Cognitive" OR "Life purpose") AND AB=("Aged" OR "Elderly" OR "Old" OR "Rural aging") AND AB=("Rural Population" OR "Communities, Rural" OR "Community, Rural" OR "Distribution, Rural Spatial" OR "Distributions, Rural Spatial" OR "Medium Communities" OR "Population, Rural" OR "Populations, Rural" OR "Rural Communities" OR "Rural Community" OR "Rural Populations" OR "Rural Settlement" OR "Rural Settlements" OR "Rural Spatial Distribution" OR "Rural Spatial Distributions" OR "Small Communities" OR "Small Community")) AND ALL=("Meta-Analysis" OR "Multicenter Study" OR"Observational Study" OR "Review" OR "Systematic Review" OR "Cross-Sectional Studies")

ALL=("Personal satisfaction" OR "Life purpose" OR "Goals" OR "Satisfaction" OR "Satisfaction, Personal" OR "Subjective Well-Being") AND ALL=("Cognition" OR "Cognitions" OR "Cognitive Function" OR "Cognitive Functions" OR "Function, Cognitive" OR "Functions, Cognitive") AND ALL=("Disability" OR "Physical functioning" OR "functional ability") AND ALL=("Aged" OR "Elderly" OR "Old" OR "Rural aging") AND ALL=("Rural Population" OR "Communities, Rural" OR "Community, Rural" OR "Distribution, Rural Spatial" OR "Distributions, Rural Spatial" OR "Medium Communities" OR "Population, Rural" OR "Populations, Rural" OR "Rural Communities" OR "Rural Community" OR "Rural Populations" OR "Rural Settlement" OR "Rural Settlements" OR "Rural Spatial Distribution" OR "Rural Spatial Distributions" OR "Small Communities" OR "Small Community")

**Scielo**

("Physical functioning" OR "Cognition" OR "Cognitions" OR "Cognitive Function" OR "Cognitive Functions" OR "Function, Cognitive" OR "Functions, Cognitive" OR "Life purpose") AND ("Aged" OR "Elderly" OR "Old" OR "Rural aging") AND ("Rural Population" OR "Communities, Rural" OR "Community, Rural" OR "Distribution, Rural Spatial" OR "Distributions, Rural Spatial" OR "Medium Communities" OR "Population, Rural" OR "Populations, Rural" OR "Rural Communities" OR "Rural Community" OR "Rural Populations" OR "Rural Settlement" OR "Rural Settlements" OR "Rural Spatial Distribution" OR "Rural Spatial Distributions" OR "Small Communities" OR "Small Community")

("Physical functioning" OR "Cognition" OR "Cognitions" OR "Cognitive Function" OR "Cognitive Functions" OR "Function, Cognitive" OR "Functions, Cognitive" OR "Life purpose") AND ("Aged" OR "Elderly" OR "Old" OR "Rural aging") AND ("Rural Population" OR "Communities, Rural" OR "Community, Rural" OR "Distribution, Rural Spatial" OR "Distributions, Rural Spatial" OR "Medium Communities" OR "Population, Rural" OR "Populations, Rural" OR "Rural Communities" OR "Rural Community" OR "Rural Populations" OR "Rural Settlement" OR "Rural Settlements" OR "Rural Spatial Distribution" OR "Rural Spatial Distributions" OR "Small Communities" OR "Small Community") AND ("Meta-Analysis" OR "Multicenter Study" OR "Observational Study" OR "Review" OR "Systematic Review")

("Physical functioning" OR "Cognition" OR "Cognitions" OR "Cognitive Function" OR "Cognitive Functions" OR "Function, Cognitive" OR "Functions, Cognitive" OR "Life purpose") AND ("Aged" OR "Elderly" OR "Old" OR "Rural aging") AND ("Rural Population" OR "Communities, Rural" OR "Community, Rural" OR "Distribution, Rural Spatial" OR "Distributions, Rural Spatial" OR "Medium Communities" OR "Population, Rural" OR "Populations, Rural" OR "Rural Communities" OR "Rural Community" OR "Rural Populations" OR "Rural Settlement" OR "Rural Settlements" OR "Rural Spatial Distribution" OR "Rural Spatial Distributions" OR "Small Communities" OR "Small Community") AND ("Meta-Analysis" OR "Multicenter Study" OR"Observational Study" OR "Review" OR "Systematic Review" OR "Cross-Sectional Studies")

((ti:("Physical functioning" OR "Cognition" OR "Cognitions" OR "Cognitive Function" OR "Cognitive Functions" OR "Function, Cognitive" OR "Functions, Cognitive" OR "Life purpose")) AND (ti:("Aged" OR "Elderly" OR "Old" OR "Rural aging")) AND (ti:("Rural Population" OR "Communities, Rural" OR "Community, Rural" OR "Distribution, Rural Spatial" OR "Distributions, Rural Spatial" OR "Medium Communities" OR "Population, Rural" OR "Populations, Rural" OR "Rural Communities" OR "Rural Community" OR "Rural Populations" OR "Rural Settlement" OR "Rural Settlements" OR "Rural Spatial Distribution" OR "Rural Spatial Distributions" OR "Small Communities" OR "Small Community"))) OR ((ab:("Physical functioning" OR "Cognition" OR "Cognitions" OR "Cognitive Function" OR "Cognitive Functions" OR "Function, Cognitive" OR "Functions, Cognitive" OR "Life purpose")) AND (ab:("Aged" OR "Elderly" OR "Old" OR "Rural aging")) AND (ab:("Rural Population" OR "Communities, Rural" OR "Community, Rural" OR "Distribution, Rural Spatial" OR "Distributions, Rural Spatial" OR "Medium Communities" OR "Population, Rural" OR "Populations, Rural" OR "Rural Communities" OR "Rural Community" OR "Rural Populations" OR "Rural Settlement" OR "Rural Settlements" OR "Rural Spatial Distribution" OR "Rural Spatial Distributions" OR "Small Communities" OR "Small Community"))) AND ("Meta-Analysis" OR "Multicenter Study" OR "Observational Study" OR "Review" OR "Systematic Review")

((ti:("Physical functioning" OR "Cognition" OR "Cognitions" OR "Cognitive Function" OR "Cognitive Functions" OR "Function, Cognitive" OR "Functions, Cognitive" OR "Life purpose") AND (ti:("Aged" OR "Elderly" OR "Old" OR "Rural aging") AND (ti:("Rural Population" OR "Communities, Rural" OR "Community, Rural" OR "Distribution, Rural Spatial" OR "Distributions, Rural Spatial" OR "Medium Communities" OR "Population, Rural" OR "Populations, Rural" OR "Rural Communities" OR "Rural Community" OR "Rural Populations" OR "Rural Settlement" OR "Rural Settlements" OR "Rural Spatial Distribution" OR "Rural Spatial Distributions" OR "Small Communities" OR "Small Community"))) OR ((ab:("Physical functioning" OR "Cognition" OR "Cognitions" OR "Cognitive Function" OR "Cognitive Functions" OR "Function, Cognitive" OR "Functions, Cognitive" OR "Life purpose") AND (ab:("Aged" OR "Elderly" OR "Old" OR "Rural aging") AND (ab:("Rural Population" OR "Communities, Rural" OR "Community, Rural" OR "Distribution, Rural Spatial" OR "Distributions, Rural Spatial" OR "Medium Communities" OR "Population, Rural" OR "Populations, Rural" OR "Rural Communities" OR "Rural Community" OR "Rural Populations" OR "Rural Settlement" OR "Rural Settlements" OR "Rural Spatial Distribution" OR "Rural Spatial Distributions" OR "Small Communities" OR "Small Community"))) AND ("Meta-Analysis" OR "Multicenter Study" OR"Observational Study" OR "Review" OR "Systematic Review" OR "Cross-Sectional Studies")

("Personal satisfaction" OR "Life purpose" OR "Goals" OR "Satisfaction" OR "Satisfaction, Personal" OR "Subjective Well-Being") AND ("Cognition" OR "Cognitions" OR "Cognitive Function" OR "Cognitive Functions" OR "Function, Cognitive" OR "Functions, Cognitive") AND ("Disability" OR "Physical functioning" OR "functional ability") AND ("Aged" OR "Elderly" OR "Old" OR "Rural aging") AND ("Rural Population" OR "Communities, Rural" OR "Community, Rural" OR "Distribution, Rural Spatial" OR "Distributions, Rural Spatial" OR "Medium Communities" OR "Population, Rural" OR "Populations, Rural" OR "Rural Communities" OR "Rural Community" OR "Rural Populations" OR "Rural Settlement" OR "Rural Settlements" OR "Rural Spatial Distribution" OR "Rural Spatial Distributions" OR "Small Communities" OR "Small Community")

**Lilacs**

("Physical functioning" OR "Cognition" OR "Cognitions" OR "Cognitive Function" OR "Cognitive Functions" OR "Function, Cognitive" OR "Functions, Cognitive" OR "Life purpose") AND ("Aged" OR "Elderly" OR "Old" OR "Rural aging") AND ("Rural Population" OR "Communities, Rural" OR "Community, Rural" OR "Distribution, Rural Spatial" OR "Distributions, Rural Spatial" OR "Medium Communities" OR "Population, Rural" OR "Populations, Rural" OR "Rural Communities" OR "Rural Community" OR "Rural Populations" OR "Rural Settlement" OR "Rural Settlements" OR "Rural Spatial Distribution" OR "Rural Spatial Distributions" OR "Small Communities" OR "Small Community") AND ( db:("LILACS"))

("Physical functioning" OR "Cognition" OR "Cognitions" OR "Cognitive Function" OR "Cognitive Functions" OR "Function, Cognitive" OR "Functions, Cognitive" OR "Life purpose") AND ("Aged" OR "Elderly" OR "Old" OR "Rural aging") AND ("Rural Population" OR "Communities, Rural" OR "Community, Rural" OR "Distribution, Rural Spatial" OR "Distributions, Rural Spatial" OR "Medium Communities" OR "Population, Rural" OR "Populations, Rural" OR "Rural Communities" OR "Rural Community" OR "Rural Populations" OR "Rural Settlement" OR "Rural Settlements" OR "Rural Spatial Distribution" OR "Rural Spatial Distributions" OR "Small Communities" OR "Small Community") AND ("Meta-Analysis" OR "Multicenter Study" OR "Observational Study" OR "Review" OR "Systematic Review") AND ( db:("LILACS"))

((ti:("Physical functioning" OR "Cognition" OR "Cognitions" OR "Cognitive Function" OR "Cognitive Functions" OR "Function, Cognitive" OR "Functions, Cognitive" OR "Life purpose")) OR (ab:("Physical functioning" OR "Cognition" OR "Cognitions" OR "Cognitive Function" OR "Cognitive Functions" OR "Function, Cognitive" OR "Functions, Cognitive" OR "Life purpose")) AND (ti:("Aged" OR "Elderly" OR "Old" OR "Rural aging")) OR (ab:("Aged" OR "Elderly" OR "Old" OR "Rural aging")) AND (ti:("Rural Population" OR "Communities, Rural" OR "Community, Rural" OR "Distribution, Rural Spatial" OR "Distributions, Rural Spatial" OR "Medium Communities" OR "Population, Rural" OR "Populations, Rural" OR "Rural Communities" OR "Rural Community" OR "Rural Populations" OR "Rural Settlement" OR "Rural Settlements" OR "Rural Spatial Distribution" OR "Rural Spatial Distributions" OR "Small Communities" OR "Small Community")) OR (ab:("Rural Population" OR "Communities, Rural" OR "Community, Rural" OR "Distribution, Rural Spatial" OR "Distributions, Rural Spatial" OR "Medium Communities" OR "Population, Rural" OR "Populations, Rural" OR "Rural Communities" OR "Rural Community" OR "Rural Populations" OR "Rural Settlement" OR "Rural Settlements" OR "Rural Spatial Distribution" OR "Rural Spatial Distributions" OR "Small Communities" OR "Small Community")) AND ("Meta-Analysis" OR "Multicenter Study" OR "Observational Study" OR "Review" OR "Systematic Review)) AND ( db:("LILACS"))

((ti:("Physical functioning" OR "Cognition" OR "Cognitions" OR "Cognitive Function" OR "Cognitive Functions" OR "Function, Cognitive" OR "Functions, Cognitive" OR "Life purpose")) OR (ab:("Physical functioning" OR "Cognition" OR "Cognitions" OR "Cognitive Function" OR "Cognitive Functions" OR "Function, Cognitive" OR "Functions, Cognitive" OR "Life purpose")) AND (ti:("Aged" OR "Elderly" OR "Old" OR "Rural aging")) OR (ab:("Aged" OR "Elderly" OR "Old" OR "Rural aging")) AND (ti:("Rural Population" OR "Communities, Rural" OR "Community, Rural" OR "Distribution, Rural Spatial" OR "Distributions, Rural Spatial" OR "Medium Communities" OR "Population, Rural" OR "Populations, Rural" OR "Rural Communities" OR "Rural Community" OR "Rural Populations" OR "Rural Settlement" OR "Rural Settlements" OR "Rural Spatial Distribution" OR "Rural Spatial Distributions" OR "Small Communities" OR "Small Community")) OR (ab:("Rural Population" OR "Communities, Rural" OR "Community, Rural" OR "Distribution, Rural Spatial" OR "Distributions, Rural Spatial" OR "Medium Communities" OR "Population, Rural" OR "Populations, Rural" OR "Rural Communities" OR "Rural Community" OR "Rural Populations" OR "Rural Settlement" OR "Rural Settlements" OR "Rural Spatial Distribution" OR "Rural Spatial Distributions" OR "Small Communities" OR "Small Community")) AND ALL("Meta-Analysis" OR "Multicenter Study" OR"Observational Study" OR "Review" OR "Systematic Review" OR "Cross-Sectional Studies") AND ( db:("LILACS"))

("Personal satisfaction" OR "Life purpose" OR "Goals" OR "Satisfaction" OR "Satisfaction, Personal" OR "Subjective Well-Being") AND ("Cognition" OR "Cognitions" OR "Cognitive Function" OR "Cognitive Functions" OR "Function, Cognitive" OR "Functions, Cognitive") AND ("Disability" OR "Physical functioning" OR "functional ability") AND ("Aged" OR "Elderly" OR "Old" OR "Rural aging") AND ("Rural Population" OR "Communities, Rural" OR "Community, Rural" OR "Distribution, Rural Spatial" OR "Distributions, Rural Spatial" OR "Medium Communities" OR "Population, Rural" OR "Populations, Rural" OR "Rural Communities" OR "Rural Community" OR "Rural Populations" OR "Rural Settlement" OR "Rural Settlements" OR "Rural Spatial Distribution" OR "Rural Spatial Distributions" OR "Small Communities" OR "Small Community") AND ( db:("LILACS"))

**PsycINFO**

"Physical functioning" *OR* **Any Field**: "Cognition" *OR* **Any Field**: "Cognitions" *OR* **Any Field**: "Cognitive Function" *OR* **Any Field**: "Cognitive Functions" *OR* **Any Field**: "Function, Cognitive" *OR* **Any Field**: "Functions, Cognitive" *OR* **Any Field**: "Life purpose" *AND* **Any Field**: "Aged" *OR* **Any Field**: "Elderly" *OR* **Any Field**: "Old" *OR* **Any Field**: "Rural aging" *AND* **Any Field**: "Rural Population" *OR* **Any Field**: "Communities, Rural" *OR* **Any Field**: "Community, Rural" *OR* **Any Field**: "Distribution, Rural Spatial" *OR* **Any Field**: "Distributions, Rural Spatial" *OR* **Any Field**: "Medium Communities" *OR* **Any Field**: "Population, Rural" *OR* **Any Field**: "Populations, Rural" *OR* **Any Field**: "Rural Communities" *OR* **Any Field**: "Rural Community" *OR* **Any Field**: "Rural Populations" *OR* **Any Field**: "Rural Settlement" *OR* **Any Field**: "Rural Settlements" *OR* **Any Field**: "Rural Spatial Distribution" *OR* **Any Field**: "Rural Spatial Distributions" *OR* **Any Field**: "Small Communities" *OR* **Any Field**: "Small Community"

"Physical functioning" *OR* **Any Field**: "Cognition" *OR* **Any Field**: "Cognitions" *OR* **Any Field**: "Cognitive Function" *OR* **Any Field**: "Cognitive Functions" *OR* **Any Field**: "Function, Cognitive" *OR* **Any Field**: "Functions, Cognitive" *OR* **Any Field**: "Life purpose" *AND* **Any Field**: "Aged" *OR* **Any Field**: "Elderly" *OR* **Any Field**: "Old" *OR* **Any Field**: "Rural aging" *AND* **Any Field**: "Rural Population" *OR* **Any Field**: "Communities, Rural" *OR* **Any Field**: "Community, Rural" *OR* **Any Field**: "Distribution, Rural Spatial" *OR* **Any Field**: "Distributions, Rural Spatial" *OR* **Any Field**: "Medium Communities" *OR* **Any Field**: "Population, Rural" *OR* **Any Field**: "Populations, Rural" *OR* **Any Field**: "Rural Communities" *OR* **Any Field**: "Rural Community" *OR* **Any Field**: "Rural Populations" *OR* **Any Field**: "Rural Settlement" *OR* **Any Field**: "Rural Settlements" *OR* **Any Field**: "Rural Spatial Distribution" *OR* **Any Field**: "Rural Spatial Distributions" *OR* **Any Field**: "Small Communities" *OR* **Any Field**: "Small Community" *AND* **Any Field**: "Meta-Analysis" *OR* **Any Field**: "Multicenter Study" *OR* **Any Field**: "Observational Study" *OR* **Any Field**: "Review" *OR* **Any Field**: "Systematic Review"

"Physical functioning" *OR* **Any Field**: "Cognition" *OR* **Any Field**: "Cognitions" *OR* **Any Field**: "Cognitive Function" *OR* **Any Field**: "Cognitive Functions" *OR* **Any Field**: "Function, Cognitive" *OR* **Any Field**: "Functions, Cognitive" *OR* **Any Field**: "Life purpose" *AND* **Any Field**: "Aged" *OR* **Any Field**: "Elderly" *OR* **Any Field**: "Old" *OR* **Any Field**: "Rural aging" *AND* **Any Field**: "Rural Population" *OR* **Any Field**: "Communities, Rural" *OR* **Any Field**: "Community, Rural" *OR* **Any Field**: "Distribution, Rural Spatial" *OR* **Any Field**: "Distributions, Rural Spatial" *OR* **Any Field**: "Medium Communities" *OR* **Any Field**: "Population, Rural" *OR* **Any Field**: "Populations, Rural" *OR* **Any Field**: "Rural Communities" *OR* **Any Field**: "Rural Community" *OR* **Any Field**: "Rural Populations" *OR* **Any Field**: "Rural Settlement" *OR* **Any Field**: "Rural Settlements" *OR* **Any Field**: "Rural Spatial Distribution" *OR* **Any Field**: "Rural Spatial Distributions" *OR* **Any Field**: "Small Communities" *OR* **Any Field**: "Small Community" *AND* **Any Field**: "Meta-Analysis" *OR* **Any Field**: "Multicenter Study" OR "Observational Study" *OR* **Any Field**: "Review" *OR* **Any Field**: "Systematic Review" *OR* **Any Field**: "Cross-Sectional Studies"

((**title**: ("Physical functioning") *OR* **title**: ("Cognition") *OR* **title**: ("Cognitions") *OR* **title**: ("Cognitive Function") *OR* **title**: ("Cognitive Functions") *OR* **title**: ("Function, Cognitive") *OR* **title**: ("Functions, Cognitive") *OR* **title**: ("Life purpose")) *OR* (**abstract**: ("Physical functioning") *OR* **abstract**: ("Cognition") *OR* **abstract**: ("Cognitions") *OR* **abstract**: ("Cognitive Function") *OR* **abstract**: ("Cognitive Functions") *OR* **abstract**: ("Function, Cognitive") *OR* **abstract**: ("Functions, Cognitive") *OR* **abstract**: ("Life purpose"))) *AND* ((**title**: ("Aged") *OR* **title**: ("Elderly") *OR* **title**: ("Old") *OR* **title**: ("Rural aging")) *OR* (**abstract**: ("Aged") *OR* **abstract**: ("Elderly") *OR* **abstract**: ("Old") *OR* **abstract**: ("Rural aging"))) *AND* ((**title**: ("Rural Population") *OR* **title**: ("Communities, Rural") *OR* **title**: ("Community, Rural") *OR* **title**: ("Distribution, Rural Spatial") *OR* **title**: ("Distributions, Rural Spatial") *OR* **title**: ("Medium Communities") *OR* **title**: ("Population, Rural") *OR* **title**: ("Populations, Rural") *OR* **title**: ("Rural Communities") *OR* **title**: ("Rural Community") *OR* **title**: ("Rural Populations") *OR* **title**: ("Rural Settlement") *OR* **title**: ("Rural Settlements") *OR* **title**: ("Rural Spatial Distribution") *OR* **title**: ("Rural Spatial Distributions") *OR* **title**: ("Small Communities") *OR* **title**: ("Small Community")) *OR* (**abstract**: ("Rural Population") *OR* **abstract**: ("Communities, Rural") *OR* **abstract**: ("Community, Rural") *OR* **abstract**: ("Distribution, Rural Spatial") *OR* **abstract**: ("Distributions, Rural Spatial") *OR* **abstract**: ("Medium Communities") *OR* **abstract**: ("Population, Rural") *OR* **abstract**: ("Populations, Rural") *OR* **abstract**: ("Rural Communities") *OR* **abstract**: ("Rural Community") *OR* **abstract**: ("Rural Populations") *OR* **abstract**: ("Rural Settlement") *OR* **abstract**: ("Rural Settlements") *OR* **abstract**: ("Rural Spatial Distribution") *OR* **abstract**: ("Rural Spatial Distributions") *OR* **abstract**: ("Small Communities") *OR* **abstract**: ("Small Community")))

(**title**: ("Rural Population") *OR* **title**: ("Communities, Rural") *OR* **title**: ("Community, Rural") *OR* **title**: ("Distribution, Rural Spatial") *OR* **title**: ("Distributions, Rural Spatial") *OR* **title**: ("Medium Communities") *OR* **title**: ("Population, Rural") *OR* **title**: ("Populations, Rural") *OR* **title**: ("Rural Communities") *OR* **title**: ("Rural Community") *OR* **title**: ("Rural Populations") *OR* **title**: ("Rural Settlement") *OR* **title**: ("Rural Settlements") *OR* **title**: ("Rural Spatial Distribution") *OR* **title**: ("Rural Spatial Distributions") *OR* **title**: ("Small Communities") *OR* **title**: ("Small Community")) *OR* (**abstract**: ("Rural Population") *OR* **abstract**: ("Communities, Rural") *OR* **abstract**: ("Community, Rural") *OR* **abstract**: ("Distribution, Rural Spatial") *OR* **abstract**: ("Distributions, Rural Spatial") *OR* **abstract**: ("Medium Communities") *OR* **abstract**: ("Population, Rural") *OR* **abstract**: ("Populations, Rural") *OR* **abstract**: ("Rural Communities") *OR* **abstract**: ("Rural Community") *OR* **abstract**: ("Rural Populations") *OR* **abstract**: ("Rural Settlement") *OR* **abstract**: ("Rural Settlements") *OR* **abstract**: ("Rural Spatial Distribution") *OR* **abstract**: ("Rural Spatial Distributions") *OR* **abstract**: ("Small Communities") *OR* **abstract**: ("Small Community"))) *AND* ((**title**: ("Aged") *OR* **title**: ("Elderly") *OR* **title**: ("Old") *OR* **title**: ("Rural aging")) *OR* (**abstract**: ("Aged") *OR* **abstract**: ("Elderly") *OR* **abstract**: ("Old") *OR* **abstract**: ("Rural aging"))) *AND* ((**title**: ("Physical functioning") *OR* **title**: ("Cognition") *OR* **title**: ("Cognitions") *OR* **title**: ("Cognitive Function") *OR* **title**: ("Cognitive Functions") *OR* **title**: ("Function, Cognitive") *OR* **title**: ("Functions, Cognitive") *OR* **title**: ("Life purpose")) *OR* (**abstract**: ("Physical functioning") *OR* **abstract**: ("Cognition") *OR* **abstract**: ("Cognitions") *OR* **abstract**: ("Cognitive Function") *OR* **abstract**: ("Cognitive Functions") *OR* **abstract**: ("Function, Cognitive") *OR* **abstract**: ("Functions, Cognitive") *OR* **abstract**: ("Life purpose"))) *AND* ((**Any Field**: ("Meta-Analysis") *OR* **Any Field**: ("Multicenter Study" *OR* "Observational Study") *OR* **Any Field**: ("Review") *OR* **Any Field**: ("Systematic Review") *OR* **Any Field**: ("Cross-Sectional Studies")))

"Personal satisfaction" *OR* **Any Field**: "Life purpose" *OR* **Any Field**: "Goals" *OR* **Any Field**: "Satisfaction" *OR* **Any Field**: "Satisfaction, Personal" *OR* **Any Field**: "Subjective Well-Being" *AND* **Any Field**: "Cognition" *OR* **Any Field**: "Cognitions" *OR* **Any Field**: "Cognitive Function" *OR* **Any Field**: "Cognitive Functions" *OR* **Any Field**: "Function, Cognitive" *OR* **Any Field**: "Functions, Cognitive" *AND* **Any Field**: "Disability" *OR* **Any Field**: "Physical functioning" *OR* **Any Field**: "functional ability" *AND* **Any Field**: "Aged" *OR* **Any Field**: "Elderly" *OR* **Any Field**: "Old" *OR* **Any Field**: "Rural aging" *AND* **Any Field**: "Rural Population" *OR* **Any Field**: "Communities, Rural" *OR* **Any Field**: "Community, Rural" *OR* **Any Field**: "Distribution, Rural Spatial" *OR* **Any Field**: "Distributions, Rural Spatial" *OR* **Any Field**: "Medium Communities" *OR* **Any Field**: "Population, Rural" *OR* **Any Field**: "Populations, Rural" *OR* **Any Field**: "Rural Communities" *OR* **Any Field**: "Rural Community" *OR* **Any Field**: "Rural Populations" *OR* **Any Field**: "Rural Settlement" *OR* **Any Field**: "Rural Settlements" *OR* **Any Field**: "Rural Spatial Distribution" *OR* **Any Field**: "Rural Spatial Distributions" *OR* **Any Field**: "Small Communities" *OR* **Any Field**: "Small Community"
