# Supplementary material for: Cognition, physical function, and life purpose in the rural elderly population: A systematic review protocol
Source: PLoS One. 2024 Jun 11;19(6):e0291699. doi: 10.1371/journal.pone.0291699 (PMC11166331; doi:10.1371/journal.pone.0291699)
Supplement: Attachment — Submitted filename: Response to reviewers.pdf [file pone.0291699.s003.pdf]

Response to reviewers

First of all, we would like to thank both reviewers for their valuable contributions to our methods article.

We would like to clarify that when we submitted the article, we noticed an error in the search date of the studies and we updated the article evaluation tool. However, despite many email exchanges with various editors of the journal, the reviewers ended up receiving the file with the error and were unable to appreciate the changes mentioned.

In the email exchanges, it was suggested that we wait for the review and submit the correct article again, which is what we are doing now, along with all the suggested corrections.

Each suggestion has been taken on board and is highlighted or commented on within the marked-up document.

Reviewer 1 mentions that the justification is partially clear and although he does not point out what needed to be made clearer, we have restructured this section. Change made on page 2 line 36 to 42.

Reviewer 1 does not approve of the methodology and although he does not point out what needed to be made clearer, we have restructured this section. However, this systematic review protocol is not based on clinical trials, but on epidemiological studies and when possible the authors have mentioned the respective prevalences. There was no sample calculation since the search was not restricted. This study includes all articles (within the inclusion criteria) that have been published up to April 2023.

We explained to Reviewers 1 and 2 that all the data used in the article will be made available as soon as it is published. We have included as an appendix at the end of the article the entire search structure that was carried out in each database according to the work of the librarian at the University of São Paulo. An extensive and very detailed search of all the possible terms used to find the articles.

Please inform proofreader 1 that the English text has been proofread and all grammatical and spelling changes are in red throughout the text.

Reviewer 1 is correct, the best instrument for this type of study is not COSMIN, this had been altered, however due to a mismatch the wrong article was sent to the reviewers without alteration. This demand has been altered on page 1 lines 16 to 17; page 4 lines 107 to 109.

Reviewer 2 suggests that the search terms be added to the article. They were added on page 7 from line 221 to the end of the text.

We are happy to answer any questions you may have.

Prof. Dr. Hércules Campos
